# Dietary Stress From Plant Secondary Metabolites Contributes to Grasshopper (*Oedaleus asiaticus*) Migration or Plague by Regulating Insect Insulin-Like Signaling Pathway

**DOI:** 10.3389/fphys.2019.00531

**Published:** 2019-05-03

**Authors:** Shuang Li, Xunbing Huang, Mark Richard McNeill, Wen Liu, Xiongbing Tu, Jingchuan Ma, Shenjin Lv, Zehua Zhang

**Affiliations:** ^1^State Key Laboratory of Biology of Plant Diseases and Insect Pests, Institute of Plant Protection, Chinese Academy of Agricultural Sciences, Beijing, China; ^2^College of Agriculture and Forestry Science, Linyi University, Linyi, China; ^3^Canterbury Agriculture and Science Centre, AgResearch, Christchurch, New Zealand; ^4^Scientific Observation and Experimental Station of Pests in Xilin Gol Rangeland, Institute of Plant Protection, Chinese Academy of Agricultural Sciences, Xilinhot, China

**Keywords:** grasshopper, diet stress, gene, plague, plant secondary metabolites

## Abstract

Diets essentially affect the ecological distribution of insects, and may contribute to or even accelerate pest plague outbreaks. The grasshopper, *Oedaleus asiaticus* B-Bienko (OA), is a persistent pest occurring in northern Asian grasslands. Migration and plague of this grasshopper is tightly related to two specific food plants, *Stipa krylovii* Roshev and *Leymus chinensis* (Trin.) Tzvel. However, how these diets regulate and contribute to plague is not clearly understood. Ecological studies have shown that *L. chinensis* is detrimental to OA growth due to the presence of high secondary metabolites, and that *S. krylovii* is beneficial because of the low levels of secondary metabolites. Moreover, in field habitats consisting mainly of these two grasses, OA density has negative correlation to high secondary metabolites and a positive correlation to nutrition content for high energy demand. These two grasses act as a ‘push-pull,’ thus enabling the grasshopper plague. Molecular analysis showed that gene expression and protein phosphorylation level of the IGF → FOXO cascade in the insulin-like signaling pathway (ILP) of OA negatively correlated to dietary secondary metabolites. High secondary metabolites in *L. chinensis* down-regulates the ILP pathway that generally is detrimental to insect survival and growth, and benefits insect detoxification with high energy cost. The changed ILP could explain the poor growth of grasshoppers and fewer distributions in the presence of *L. chinensis*. Plants can substantially affect grasshopper gene expression, protein function, growth, and ecological distribution. Down-regulation of grasshopper ILP due to diet stress caused by high secondary metabolites containing plants, such as *L. chinensis*, results in poor grasshopper growth and consequently drives grasshopper migration to preferable diet, such as *S. krylovii*, thus contributing to grasshopper plague outbreaks.

## Introduction

Nearly half of all insect species are herbivores (Gatehouse, 2002; Wu and Baldwin, 2010), that have co-evolved with plants for 350 million years (Kessler and Baldwin, 2002). Phytophagous insects have specific adaptability to various host plants (Howe and Jander, 2008; Rominger et al., 2009; Unsicker et al., 2010), which also determine their ecological distribution and population dynamics (Whitman, 1990; Franzke et al., 2010). Such phytophagous insect-host plant relationships are examples of co-adaptation, co-evolution, and co-speciation (Scriber, 2002; Powell et al., 2006; Zhang and Fielding, 2011).

Plants have evolved various defense mechanisms against phytophagous insect (Padul et al., 2012). Such defenses can be broadly classified into two categories: constitutive defenses, including physiological barrier and nutritional hurdle; and inducible defenses, including secondary metabolites and protein inhibitors (PIs) (Zavala et al., 2004; Wu and Baldwin, 2010; Padul et al., 2012). Both types are achieved through similar means but differ in that constitutive defenses are present before herbivore attacks, while induced defenses are activated only when attacks occur (Wu and Baldwin, 2010; Padul et al., 2012). The regulatory elements in networks that modulate herbivory induced responses in plants mainly include jasmonic acid (JA), salicylic acid (SA), and ethylene (ET) (Baldwin, 1998; Wu and Baldwin, 2010; Kessler and Baldwin, 2002). Plant defense mechanisms are also categorized into direct and indirect responses according to their role and function (Wu and Baldwin, 2010). Indirect defenses include volatile organic compounds produced when the plant is subject to herbivory that attract predators and parasitoids of the insect (Dicke and Loon, 2000; Despland and Simpson, 2005; Despres et al., 2007). Plant chemistry, especially those associated with secondary metabolites, is an important component of the phenotype that mediates plant-insect interactions (Mendelsohn and Balick, 1995; Despres et al., 2007). In general, insect dietary stress predominantly originates from high levels of plant secondary metabolites (Behmer, 2009; Despres et al., 2007; Wetzel et al., 2016).

Conversely, insect herbivores also evolved various detoxification mechanisms, mainly including avoidance, excretion, sequestration, metabolic resistance, and target mutation, which allow them to consume and develop on toxic plants producing high levels of secondary metabolites (Despres et al., 2007). Such insect feeding continues the selective pressure on plants to develop increased or novel chemical defenses (Musser et al., 2002; Becerra, 2003; Dussourd, 2003; Helmus and Dussourd, 2005). Insect herbivores’ response to diet stress are well-documented in many insects, mainly focusing on insect behavioral, physiological, chemical, genetic, ecological, and evolutionary mechanisms (Raubenheimer and Simpson, 2004; Giri et al., 2006; Dicke and Baldwin, 2010; Ibanez et al., 2013). Some herbivorous species are strongly attractive or indispensable for some specific plant species (Schutz et al., 1997). Existence of those plants may contribute to, or even accelerate, insect population outbreaks (Powell et al., 2006). For example, *Phragmites australis* (Cav.) Trin. provides an optimal food source (Zhu, 2004; Ji et al., 2007), which could significantly benefit *Locusta migratoria manilensis* (Meyen) population growth.

**FIGURE 1:**
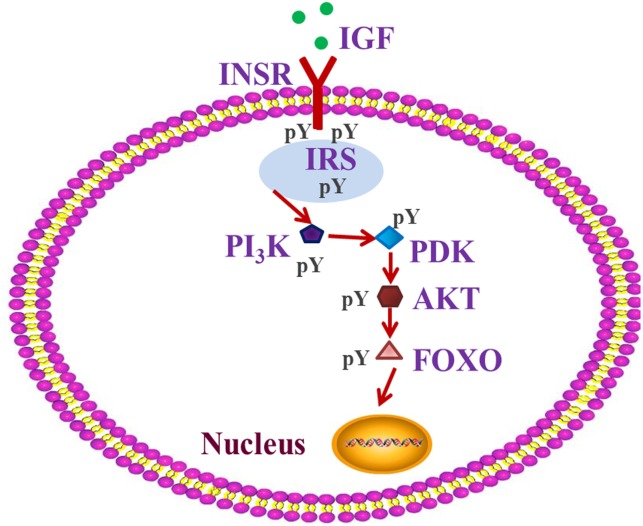
IGF-PI_3_K-AKT-FOXO pathway of insulin signaling. The insulin-like signaling system includes different well-defined ligands, such as insulin-like growth factor (IGF), which regulate the activity of the homologous insulin receptor INSR. Insulin receptor substrate (IRS) proteins act as messenger molecule-activated receptors to signaling, and which is an important step in insulin’s action. Phosphoinositide 3-kinase (PI_3_K), 3-phosphoinositide-dependent protein kinase (PDK), and protein kinase B (AKT), three major nodes downstream of IRS, and have been implicated in many of the metabolic actions of insulin. The forkhead transcription factor (FOXO) regulates transcription of genes involved in stress resistance, xenobiotic detoxification and DNA repair. FOXO is negatively regulated by insulin-like signaling when the PI_3_K → AKT cascade stimulates phosphorylation of FOXO and promotes its secretion from the nucleus and inactivation in the cytosol.

Many genes and related pathways of insects, such as the insect insulin-like signaling pathway (ILP), play important roles in specific insect-diet relationship, and contribute to the variation of insect performance (Bishop and Guarente, 2007; Taguchi and White, 2008; Ragland et al., 2015). The insect ILP is considered to act as a sensor of the dietary status and to stimulate the progression of anabolic events when the status is positive (Taguchi and White, 2008; Badisco et al., 2013). It plays a crucial role in a number of fundamental and interrelated physiological processes, including insect growth, energy metabolism, and detoxification (Claeys et al., 2002; Wu and Brown, 2006; Kawada et al., 2010; Fujisawa and Hayakawa, 2012). Many studies in different metazoan species have indeed demonstrated that not only the insulin-related peptides are evolutionarily conserved, but also the components of their signaling pathway, such as IGF/INSR/IRS/PI_3_K/PDK/AKT/FOXO (Figure 1), which play an important role in insulin resistance, are also conserved (Sim and Denlinger, 2008; Badisco et al., 2013). Insulin signaling can be delivered by phosphorylation or dephosphorization of proteins, such as INSR/IRS/AKT/FOXO (Kramer et al., 2008; Hedrick, 2009). ILP changes associated with diet stress can influence insect growth (Kawada et al., 2010; Fujisawa and Hayakawa, 2012; Badisco et al., 2013), which can potentially affect pest distribution or even plague outbreaks. However, the role of the ILP signaling pathway in regulating pest plague outbreaks is poorly understood.

*Oedaleus asiaticus* Bey-Bienko is a specialist grass-feeder, with preference for Poaceae species, particularly *Stipa krylovii* Roshev (Poaceae) (Zhang et al., 2013; Qin et al., 2017). It is a member of the subfamily Oedipodinae (Orthoptera: Acrididae: Oedipodinae), and a dominant grasshopper of northern Asian grasslands, generally distributed in Inner Mongolia of north China (Cease et al., 2012; Zhang et al., 2014). Outbreaks of *O. asiaticus* have often lead to significant loss in grasses and economic disruption (Liu et al., 2013). From routine surveys of plant and grasshoppers composition in *Stipa* (*S. krylovii*) and *Leymus* (*Leymus chinensis*) (Trin.) Tzvelev (Poales: Poaceae) grasslands (Han et al., 2008), we found that *O. asiaticus* was mainly confined to the former (Huang et al., 2016, 2017a). In addition, we also found that the presence of plant secondary metabolites in *L. chinensis* can have a negative impact on *O. asiaticus* growth parameters (Huang et al., 2017a), while acting as a catalyst to drive grasshopper migration and plague outbreak. In the present study, we investigated how diet stress influences insect growth, distribution, and the ILP, to decipher the relationship between diet stress and pest outbreaks. We also discuss the role of diet stress in driving pest plague outbreaks, and how this information provides new insights into pest management.

## Materials and Methods

### Ethics Statement

Insects (*O. asiaticus*) were collected from the Xilin Gol grassland from 2011 to 2017. It is a common agricultural pest and not in the “List of Protected Animals in China.” No permits were required for the described field studies.

### Study Area

The research grassland (E115°13′–117°06′, N43°02′–44°52′) is located in the Xilin Gol League, Inner Mongolia, northeast China. This grassland is a region representative of the Eurasian steppe grassland and characterized by *L. chinensis*- and *S. krylovii* -dominated plant communities (Han et al., 2008). The above-ground biomass of these two plants accounts for more than 80% of the total community production (Huang et al., 2016). Plants in this grassland cover only 30 to 40% of the ground area with the remainder being bare ground for rapid steppe degradation in part driven by livestock over-grazing (Chen and Wang, 2000; Han et al., 2008). *O. asiaticus* is the dominante grasshopper species (Guo et al., 2006), and generally hatches between late-May and late-June, reaching adulthood between early to late July (Huang et al., 2016). This grasshopper mainly feeds on Poaceae species, particularly *S. krylovii* (Wu et al., 2012; Zhang et al., 2013).

### Large-Scale Survey of Plant Biomass and *O. asiaticus* Density on Grazed Grassland

We examined the relationship between above-ground plant biomass composition and *O. asiaticus* density in this grazed grassland area, in mid-July for each year from 2011 to 2017. Those areas were mainly dominated by *S. krylovii* and *L. chinensis*, with rare distribution of other plant species. Each year, we selected eight 1 km^2^ sample plots (∼10 km apart). In each plot, we selected five 1-m^2^ quadrats (∼50 m apart) randomly. Grass *S. krylovii* and *L. chinensis* plants within each quadrat were cut to ground level and placed separately into envelopes. Those collected grass were dried at 90°C for 24 h, and weighed to provide the relative mean above ground biomass (g DM/m^2^) of the two plant species for each plot.

We estimated the relative density of the grasshopper *O. asiaticus* in each plot in mid-July for each year 2011 to 2017, using the same method described in our previous published paper (Huang et al., 2016). We averaged the four samples in each plot, to derive a relative *O. asiaticus* density (number of individuals per 100 sweep-nets) for each of the eight plots in each year.

Sampling produced eight means (one for each of the eight 1 km^2^ plots) for both grass species and *O. asiaticus* relative densities for each year from 2011 to 2017.

### Cage Study of *O. asiaticus* Growth in Grassland

To study *O. asiaticus* growth for different host plant species, a field cage study was carried out on *S. krylovii* and *L. chinensis* grasslands during late June of both 2016 and 2017. In each of those two grasslands, all of the other plants were removed to assure that only one host plant remained. We constructed 10 screen cages (1 m × 1 m × 1 m) using iron rod frames covered with 1 mm^2^ cloth mesh. In each plant species, five cages were used as the biological replicates. All of the visible spiders and other natural enemies in the field cages were removed carefully before adding female 4th instar *O. asiaticus*.

We collected 4th instar *O. asiaticus* nymphs by sweep net from the grassland mainly containing these two grasses on 21 June, 2016 and 2017. Those collected individuals were then temporarily maintained in metal-frame cages and starved for 24 h. Then, female 4th instar *O. asiaticus* nymphs (total 160 individuals) were assigned to the 10 field cages (16 individuals per cage) randomly. Those experimental individuals were selected to be as uniform in size as possible, with fresh body mass weighed and verified by ANOVA to confirm there were no significant differences in the weight of *O. asiaticus* nymphs amongst the four treatments. Besides, we killed another cohort of 30 *O. asiaticus* 4th instar females by chloroform and dried them at 90°C for 24 h. Those dried grasshoppers were individually weighed (mg), and a mean dry mass determined to serve as the baseline data. Once grasshoppers were assigned to every cage, we checked field cages to monitor survival every day. In each cage, grasshoppers were able to feed *ad libitum* on grass. The plant biomass in the cages could provide sufficient food to allow development through to adults. When all of the surviving individuals became female adults, they were also killed and dried using the same method above to get adult dry mass (mg). Adult body dry mass was used to calculate grasshopper increased body mass (mg) by subtracting the 4th instar body dry mass. Grasshopper survival rate (%) was calculated by the number of adult individuals / number of initial individuals (*n* = 16). Grasshopper development time (days) was calculated by the following formula: $DT=\frac{\sum_{i=1}^{n}i^{*}N_{i}}{N_{t}}$, where *i* is the number of days from 4th instar to adult; *N*_i_ is the number of individuals with the development time corresponding to the value of “*i*”; and *N*_t_ is the number of adult individuals (Huang et al., 2016). Grasshopper growth rate (mg/day) was calculated by increased body dry mass / development time, and overall performance calculated from growth rate × survival rate (Cease et al., 2012).

### Grass Chemical Traits

In 2017, we cut the remaining plants from each cage at ground level after removing the adults. Each species were placed in a separate plastic container and used to detect the starch, nitrogen, and lipid content by Iodine-starch colorimetric method, Kjeldahl method, and Soxhlet extraction method, respectively. We then estimated crude protein content of each plant sample by 6.25 × nitrogen content (Bawa and Yadav, 1986). We also detected plant secondary compounds (tannins, phenols, alkaloids, terpenoids, and flavonoids) content in each sample by high performance liquid chromatography (HPLC), using the same method described in previous published papers (Ossipov et al., 1995; Griffin et al., 1999; Naczk, 2004; Friedman et al., 2006; Guo, 2007).

### Gene Expression of ILP

We investigated seven genes of *O. asiaticus* ILP signaling pathway (*IGF*, *INSR*, *IRS*, *PI3K*, *PDK*, *AKT*, and *FOXO*) to compare their relative expression when exposed to different plant species. Unigene sequences were acquired from our previous transcriptome profiles (RSA accession number SRP072969) to design gene-specific primers (Supplementary Table S1). We randomly collected one adult sample from each replicate of the two treatments (10 samples). The relative expression of these genes was analyzed by qRT-PCR, using the same method described in our previous published paper (Huang et al., 2017b). Then, relative gene expression was normalized by the internal standard (actin), and calculated using the 2^-ΔΔCT^ Method. Expression values were adjusted by setting the expression of *O. asiaticus* feeding on *S. krylovii* to be one for each gene. All qRT-PCRs for each gene with 10 samples (five biological replicates for each treatment) used 3 technical replicates per experiment.

### Protein Phosphorylation Analysis

We used the rapid ELISA-Based Measurement to detect the protein phosphorylation of IRS, INSR, AKT, and FOXO in the insect ILP. Grasshopper samples were homogenized in 1 ml PBS, and the resulting suspension subjected to ultrasonication to further break the cell membranes. After that, we centrifuged the homogenates for 15 min at 5000 rpm, collected the supernatants and stored at -20°C until required for further analysis.

We prepared all of the reagents (Supplementary Table S2) and brought all of the reagents and samples to room temperature (18°C-25°C). After 30 min at room temperature, we added 50 μl standard to each standard well, 50 μl sample to each sample well and 50 μl sample diluent to each blank/control well. Then, 100 μl of HRP-conjugate reagent was added to each well, and covered with an adhesive strip and incubated for 60 min at 37°C. The Microtiter Plate was washed 4 times using Wash Buffer (undiluted), and then 50 μl Chromogen Solution A and 50 μl Chromogen Solution B was sequentially added to each well. This was then gently mixed and then incubated for 15 min at 37°C in dark, after which 50 μl Stop Solution was added to each well. A change in the color of the solution from blue to yellow was expected. If the color in the wells was green or the color change did not appear uniform, the plate was gently taped to ensure thorough mixing. The Optical Density (O.D.) at 450 nm was read using a Microelisa Stripplate reader within 15 min of adding the Stop Solution. Then, we constructed the standard curve of each protein, and calculated the amount of phosphorylated protein in each sample.

### Data Analysis

Student’s *t* test was used to compare grasshopper growth variables (body size, survival rate, development time, growth rate, and overall performance), protein phosphorylation level, and relative gene expression. Linear regression was used to analyze the growth, distribution, ILP gene expression, and phosphorylation to grass chemical content in *O. asiaticus*. We used SAS version 8.0 for these analyses.

## Results

### Chemical Traits of *Stipa krylovii* and *Leymus chinensis* Grasses

**FIGURE 2:**
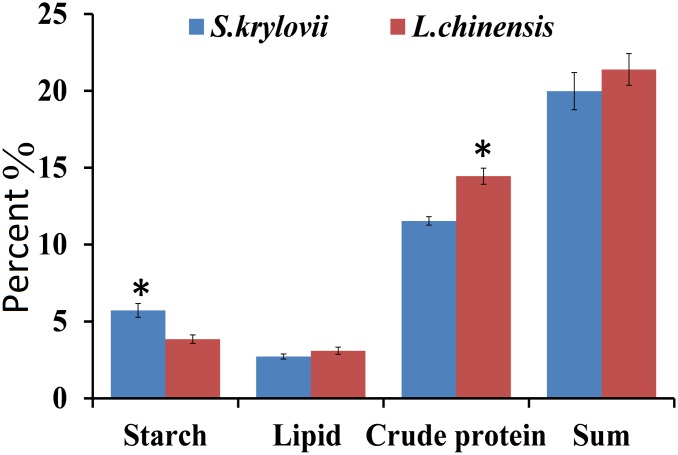
Percentage (±SD, %) of nutrition components (starch, lipid, and crude protein) in the grass species *L. chinensis* and *S. krylovii*. ^∗^indicates *P* < 0.05 (*t* test).

**FIGURE 3:**
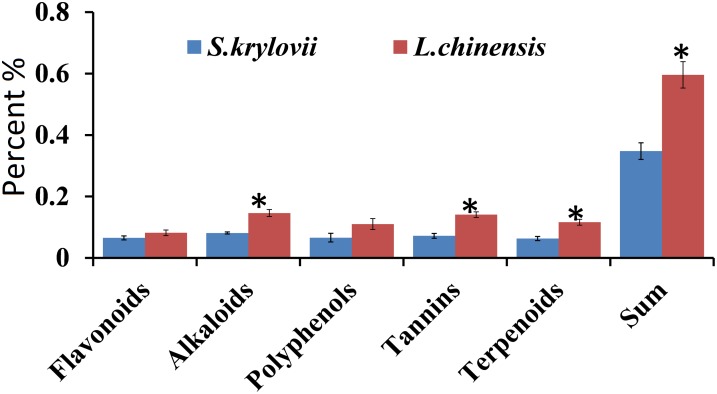
Percentage (±SD, %) of secondary metabolites (terpenoids, tannins, phenols, alkaloids, and flavonoids) in the grass species *S. krylovii* and *L. chinensis*. ^∗^indicates *P* < 0.05 (*t* test).

The main nutrition and secondary metabolites were different in the two plant species (Figure 2, 3). The highest starch content (*t* = 3.805, *df* = 8, *P* = 0.005) was present in *S. krylovii*, and the highest crude protein content (*t* = 4.085, *df* = 8, *P* = 0.004) was present in *L. chinensis* (Figure 2). The sum of the three nutritive substances (crude protein, lipid, and starch) was not significantly different between the two species.

For the five secondary metabolites, *L. chinensis* had higher levels of alkaloids (*t* = 9.440, *df* = 8, *P* < 0.001), tannins (*t* = 8.534, *df* = 8, *P* < 0.001), and terpenoids (*t* = 8.149, *df* = 8, *P* < 0.001) than *S. krylovii*. The total amount of all five secondary metabolites was highest in *L. chinensis* (*t* = 11.099, *df* = 8, *P* < 0.001) (Figure 3).

### Relationship Between *O. asiaticus* Performance and Grass Chemical Composition

**FIGURE 4:**
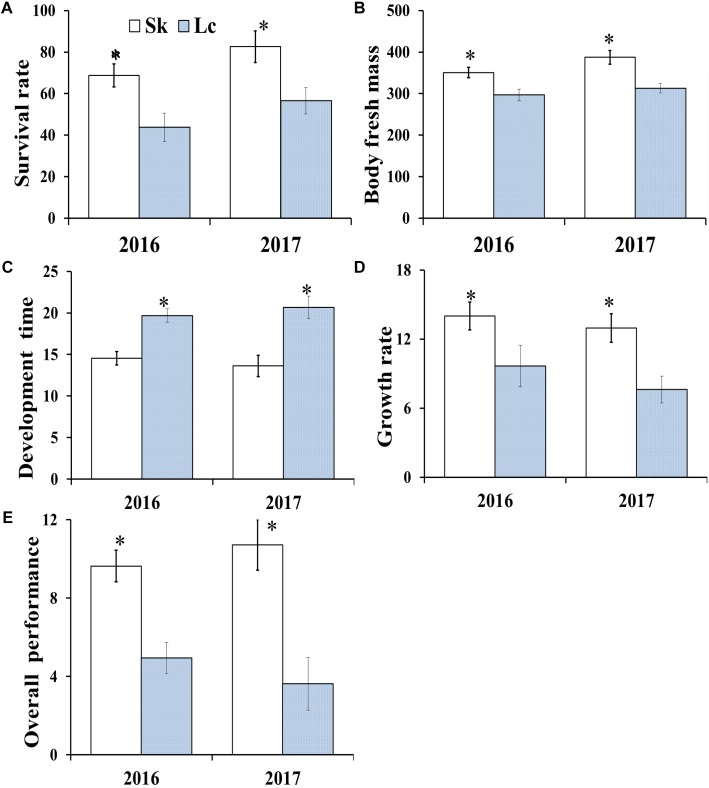
**(A)** Mean survival rate of *O. asiaticus* from fourth instar to adult, **(B)** mean dry mass (mg) of adults, **(C)** mean developmental time (days) from fourth instar to adult, **(D)** growth rate (mg/day), and **(E)** overall performance when fed on *S. krylovii* (Sk) or *L. chinensis* (Lc). Data are mean ± SD. ^∗^indicates *P* < 0.05 (*t* test).

**FIGURE 5:**
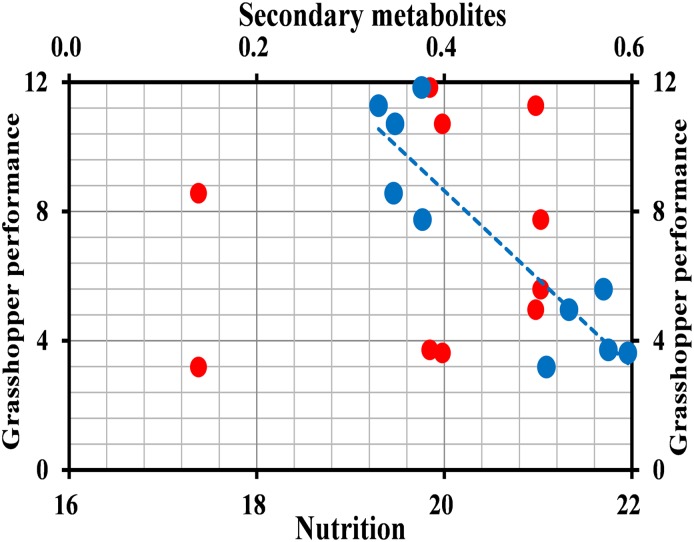
Relationship between grasshopper overall performance and plant chemical composition. Red circles indicate total nutrition, and blue triangles indicate total secondary metabolites.

The mean survival rate (Figure 4A) (2016: *t* = 7.732, *df* = 8, *P* < 0.001; 2017: *t* = 6.641, *df* = 8, *P* < 0.001), developmental time (*t* = 4.647, *df* = 8, *P* = 0.002; *t* = 5.077, *df* = 8, *P* = 0.001) (Figure 4B), adult fresh mass (*t* = 10.521, *df* = 8, *P* < 0.001; *t* = 13.311, *df* = 8, *P* < 0.001) (Figure 4C), growth rate (*t* = 7.838, *df* = 8, *P* < 0.001; *t* = 13.311, *df* = 8, *P* < 0.001) (Figure 4D), and overall performance (*t* = 7.732; *df* = 8; *P* < 0.001; *t* = 5.949; *df* = 8; *P* < 0.001) (Figure 4E) of *O. asiaticus* were significantly lower in insects that fed on *L. chinensis*, compared to those that fed on *S. krylovi.* Feeding on *L. chinensis* provided less benefit for *O. asiaticus* growth and development. There was a significant negative linear relationship between grasshoppers and total plant secondary metabolites (Figure 5; *R*^2^ = 0.893, *F* = 76.095, *P* < 0.001). Based on these results, we concluded that *S. krylovi* with less secondary metabolites resulted in better grasshopper growth. Conversely, high levels of secondary metabolites in *L. chinensis*, created a higher level of dietary stress, which lowered growth of *O. asiaticus*.

### Relationship Between *O. asiaticus* Density and Grass Chemical Traits in Field Habitat

**FIGURE 6:**
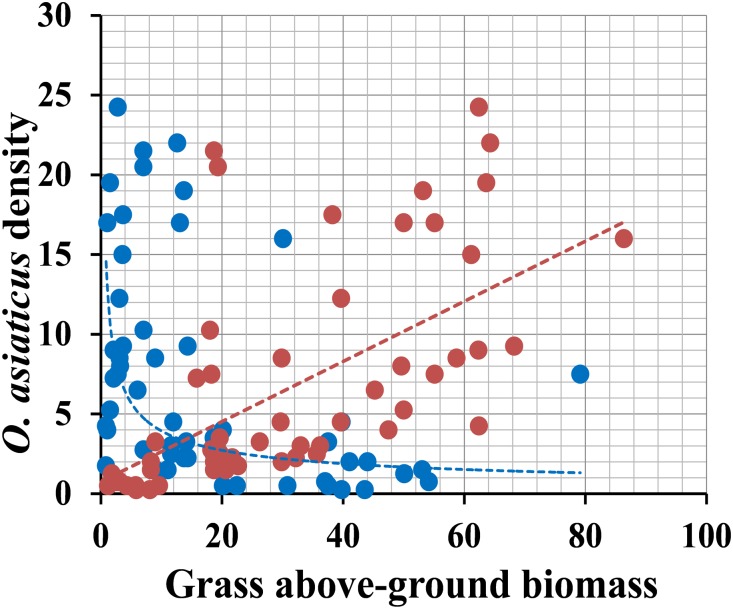
Relationship between the relative density of *O. asiaticus* (mean number of individuals per 100 sweep-nets) and mean above-ground biomass (g/m^2^) of *S. krylovii* (red circles) and *L. chinensis* (blue circles). Data combined from measurements recorded from 2011 – 2017 (*N* = 8 means per year), with 2011–2014 values from our published data.

Survey results of 7 years showed that the relative density of *O. asiaticus* exhibited a significant positive relationship to *S. krylovii* above-ground biomass (Figure 6, linear correlation: y = 0.1886x + 0.7506, *R*^2^ = 0.365, *N* = 56, *F* = 31.006, and *P* < 0.001). In contrast, the relative density of *O. asiaticus* was significant negative related to *L. chinensis* above-ground biomass (Figure 6, power correlation: *y* = 13.393x^-0.523^, *R*^2^ = 0.283, *N* = 56, *F* = 21.298, and *P* < 0.001).

**FIGURE 7:**
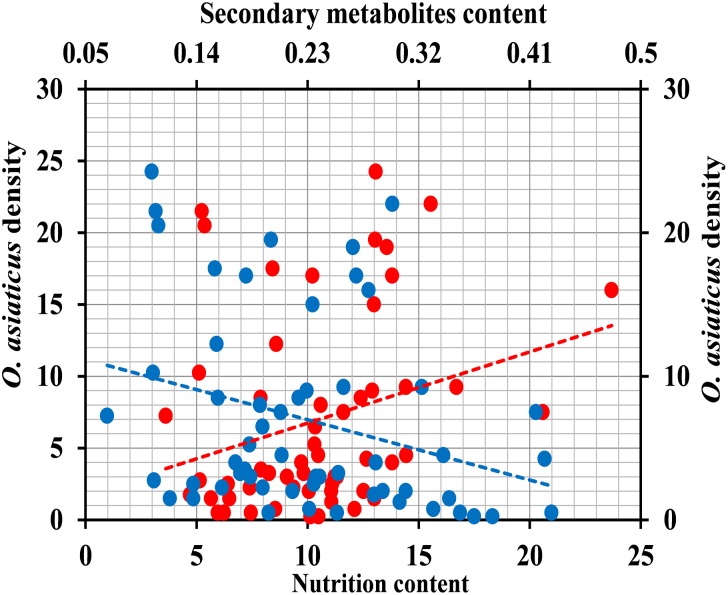
Relationship between relative density of grasshopper *O. asiaticus* (mean number of individuals per 100 sweep-nets) and chemical traits (g/m^2^). Red circles indicate total nutrition, and blue circles indicate total secondary metabolites.

We also used above plant chemical data to evaluate total chemical traits of surveyed grassland. There was significant multiple linear relationship between grasshopper density and variable nutrition (x_1_) and secondary metabolites (*x*_2_) (Figure 7; *y* = 1.11*x*_1_ - 49.435*x*_2_ + 6.687, *R*^2^ = 0.366, *F* = 15.285, and *P* < 0.001). From the standardization regression coefficient (*x*_1_ = 0.638, *x*_2_ = 0.624), we concluded that the content of secondary metabolites had the largest effect (negative) on grasshopper density.

### Change in *O. asiaticus* ILP Due to Diet Stress

#### Gene Expression

**FIGURE 8:**
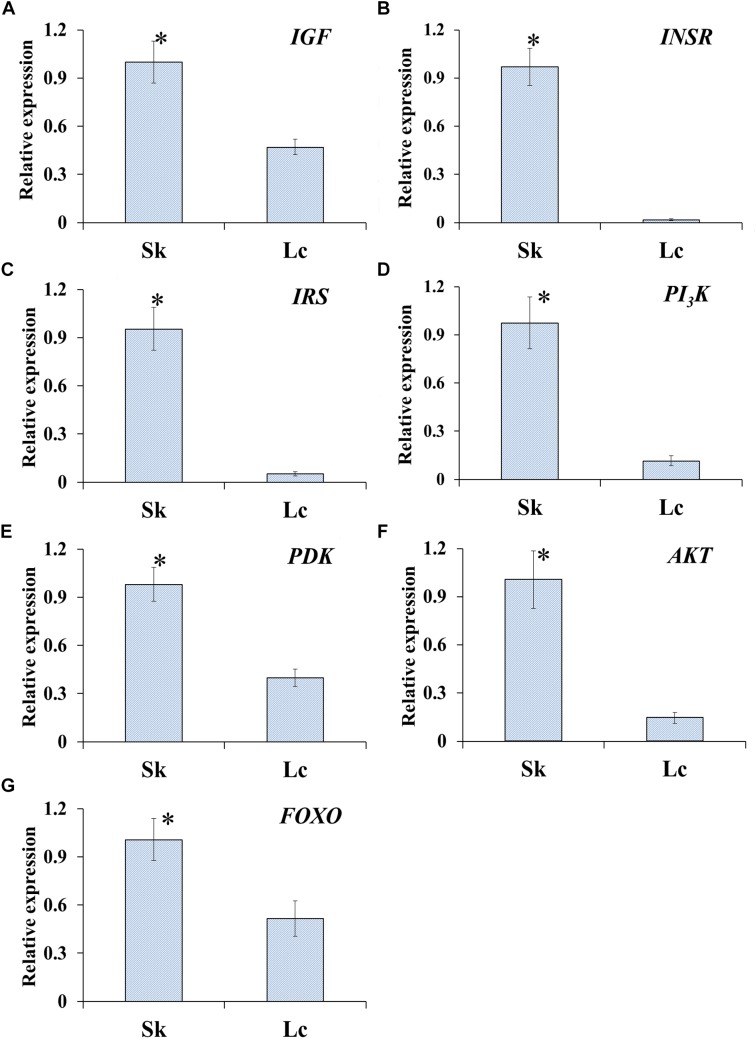
Relative expression (±SD) of seven genes of ILP in grasshopper, *O. asiaticus*, that fed on *S. krylovii* (Sk), and *L. chinensis* (Lc). ^∗^indicates *P* < 0.05 (*t* test). *IGF* **(A)**, insulin-like growth factor; *INSR* **(B)**, homologous insulin receptor; *IRS* **(C)**, insulin receptor substrate; *PI_3_K* **(D)**, phosphoinositide 3-kinase; *PDK* **(E)**, 3-phosphoinositide-dependent protein kinase; *AKT* **(F)**, protein kinase B; and *FOXO* **(G)**, forkhead transcription factor.

**FIGURE 9:**
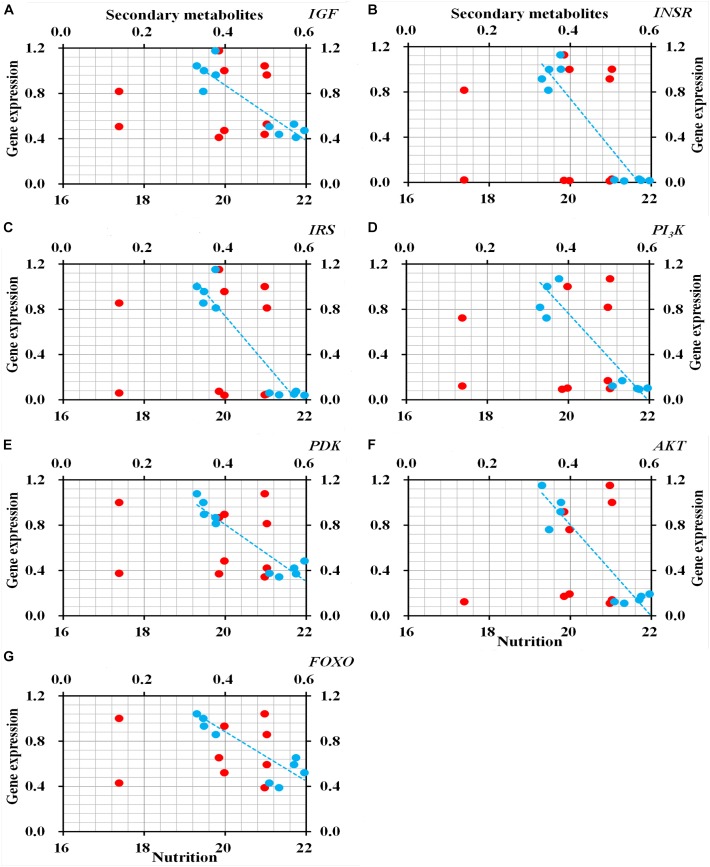
Relationship between relative gene expression in grasshopper and chemical traits in plants (%). Red circles indicate total nutrition, and blue circles indicate total secondary metabolites. *IGF* **(A)**, insulin-like growth factor; *INSR* **(B)**, homologous insulin receptor; *IRS* **(C)**, insulin receptor substrate; *PI_3_K* **(D)**, phosphoinositide 3-kinase; *PDK* **(E)**, 3-phosphoinositide-dependent protein kinase; *AKT* **(F)**, protein kinase B; and *FOXO* **(G)**, forkhead transcription factor.

qRT-PCR to determine the relative expression of seven genes in ILP signaling pathway indicated that the genes *IGF* (*t* = 8.472, *df* = 8, *P* < 0.001), *INSR* (*t* = 17.851, *df* = 8, *P* < 0.001), *IRS* (*t* = 14.951, *df* = 8, *P* < 0.001), *PI_3_K* (*t* = 14.951, *df* = 8, *P* < 0.001), *PDK* (*t* = 8.944, *df* = 8, *P* < 0.001), *AKT* (*t* = 10.633, *df* = 8, *P* < 0.001), and *FOXO* (*t* = 6.529, *df* = 8, *P* < 0.001) were significantly down-regulated in *O. asiaticus* that fed on *L. chinensis* (Figure 8). Grass secondary metabolites also exhibited a significant negative relationship (Figure 9) to the gene expression of *IGF* (*R*^2^ = 0.832, *F* = 39.626, *P* < 0.001), *INSR* (*R*^2^ = 0.890, *F* = 65.039, *P* < 0.001), *IRS* (*R*^2^ = 0.893, *F* = 76.095, *P* < 0.001), *PI_3_K* (*R*^2^ = 0.810, *F* = 34.016, *P* < 0.001), *PDK* (*R*^2^ = 0.884, *F* = 60.782, *P* < 0.001), *AKT* (*R*^2^ = 0.866, *F* = 60.123, *P* < 0.001), and *FOXO* (*R*^2^ = 0.720, *F* = 20.565, *P* = 0.002). Based on this evidence, we concluded that *S. krylovi* with less secondary metabolites determined the high gene expression involved in grasshopper ILP. But, *L. chinensis* with high secondary metabolites, induced dietary stress and down-regulated gene expression in the grasshopper.

#### Protein P- Level of ILP

**FIGURE 10:**
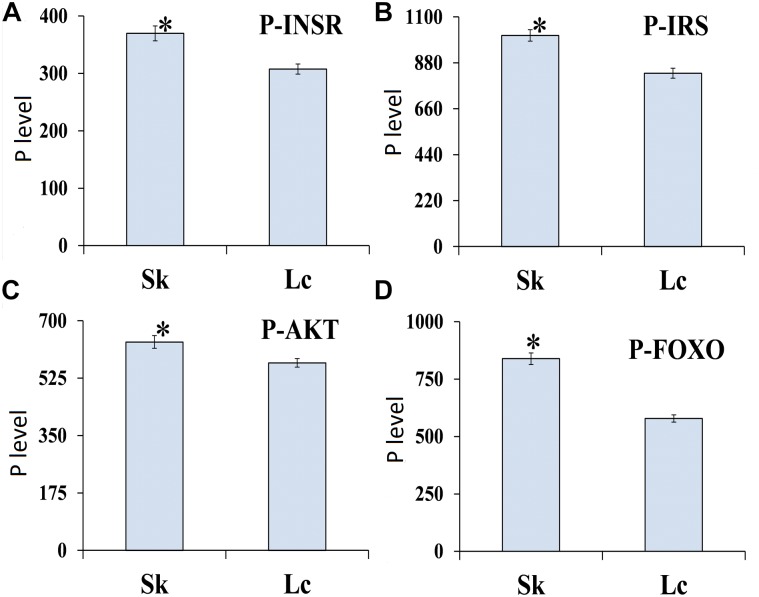
Phosphorylation level (pg/g) of four proteins of ILP in *O. asiaticus* that fed on *S. krylovii* (Sk) and *L. chinensis* (Lc). ^∗^indicates *P* < 0.05 (*t* test). P-INSR **(A)**, Phosphorylated homologous insulin receptor; P-IRS **(B)**, Phosphorylated insulin receptor substrate; P-AKT **(C)**, Phosphorylated protein kinase B; and P-FOXO **(D)**, Phosphorylated forkhead transcription factor.

**FIGURE 11:**
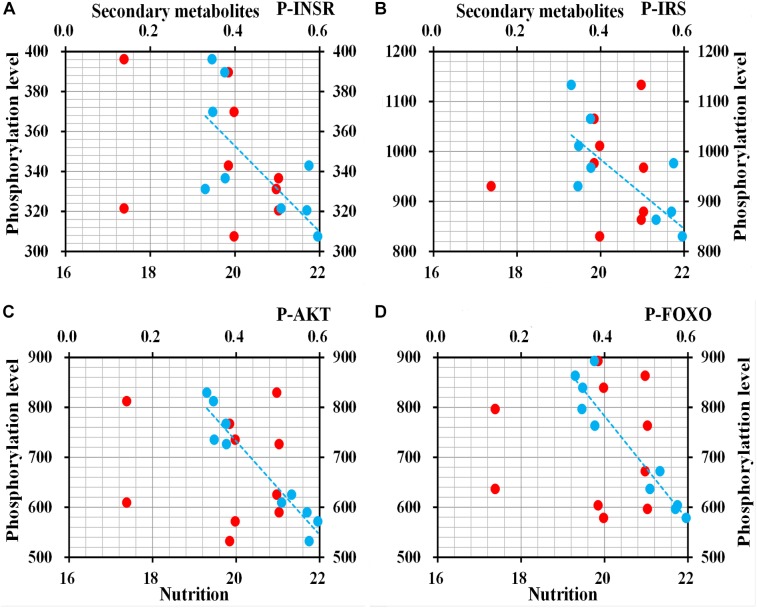
Relationships between protein phosphorylation level (pg/g) of ILP and grass chemical traits in plants (%). Red dots indicate total nutrition; blue dots indicate total secondary metabolites. P-INSR **(A)**, Phosphorylated homologous insulin receptor; P-IRS **(B)**, Phosphorylated insulin receptor substrate; P-AKT **(C)**, Phosphorylated protein kinase B; and P-FOXO **(D)**, Phosphorylated forkhead transcription factor.

ELISA to determine the phosphorylation level of four proteins in ILP signaling pathway indicated that INSR (*t* = 3.0269, *df* = 8, *P* = 0.016), IRS (*t* = 3.247, *df* = 8, *P* = 0.012), AKT (*t* = 7.237, *df* = 8, *P* < 0.001), and FOXO (*t* = 7.498, *df* = 8, *P* < 0.001) were phosphorylated at the lowest levels in *O. asiaticus* fed on *L. chinensis* (Figure 10). Grass secondary metabolites exhibited a significant negative relationship to phosphorylation levels of INSR (*R*^2^ = 0.462, *F* = 6.811, *P* = 0.03), IRS (*R*^2^ = 0.509, *F* = 8.212, *P* = 0.021), AKT (*R*^2^ = 0.925, *F* = 98.120, *P* < 0.001), and FOXO (*R*^2^ = 0.901, *F* = 72.656, *P* < 0.001) (Figure 11). Based on these results, we concluded that low levels of secondary metabolites produced by *S. krylovi* determined the high protein phosphorylation level of proteins involved in ILP. But high levels of secondary metabolites found in *L. chinensis*, acted as a dietary stress, and down-regulated protein phosphorylation level.

## Discussion

Migration or plague of grasshoppers generally can cause massive agricultural damage, and lead to tremendous economic losses (Stige et al., 2007; Liu et al., 2013). To achieve the control of pest species, it is essential to understand the factors that lead to migration and plague outbreaks. Plant species in the grassland could significantly influence population dynamics and spatial distribution of grasshoppers (Unsicker et al., 2010; Masloski et al., 2014). Some plant species are strong attractive or indispensable to some specific herbivore species, and may contribute to or even accelerate pest plague outbreaks or migration (Schutz et al., 1997; Scriber, 2002; Powell et al., 2006). Understanding the relationship of grasshoppers with their host plant species has great significance for improving management strategies (Cease et al., 2012; Huang et al., 2016).

Interactions between plants and insects are among the closest and most dynamic ecological relationships in nature, with both taxa exerting mutual effects on one another. Such relationships can vary from beneficial to detrimental, as observed in both *S. krylovii* and *L. chinensis* in our present and previous studies (Huang et al., 2016, 2017a). Both *S. krylovii* and *L. chinensis* are dominant and widely distributed grasses across Inner Mongolia grasslands, with their above-ground biomass accounting for the most of the total community production (Huang et al., 2016). Interestingly, these two grasses had opposite roles in grasshopper migration and plague. Grasshoppers that fed on *L. chinensis* had reduced growth variables (size, growth rate, development, and survival) compared to those fed on *S. krylovii*, which indicated that *L. chinensis* was unsuitable for *O. asiaticus* compared to *S. krylovii*. These results are consistent with previous studies (Wu et al., 2012; Zhang et al., 2013), which also indicated that *S. krylovii* was the best food resource and a preferred plant host for *O. asiaticus* grasshopper. In the field, we also found that dry matter consumption and loss was highest for *S. krylovii* and that grasshoppers generally avoided *L. chinensis*.

In the present study, 7 years of extensive monitoring also showed that *O. asiaticus* density positively correlated with the above-ground biomass of *S. krylovii*, and negatively correlated with *L. chinensis* above-ground biomass. Grasshopper *O. asiaticus* mainly distributed in *S. krylovii* dominated grassland, with a lower distribution in *Leymus*-dominated grassland, a relationship also reported by other researchers, who found that grasshopper plague outbreaks usually occurred in *Stipa*-dominated grasslands (Cease et al., 2012). Wu et al. (2012) used redundancy analysis and Huang et al. (2015) used the projection pursuit model and showed that the existence of *S. krylovii* positively affected *O.asiaticus* density in Inner Mongolia grassland, while *L. chinensis* had a negative effect on *O. asiaticus* density. Based on the perspective of grasshopper biology, *S. krylovii* is the favorable host plant while *L. chinensis* is a detrimental host plant.

**FIGURE 12:**
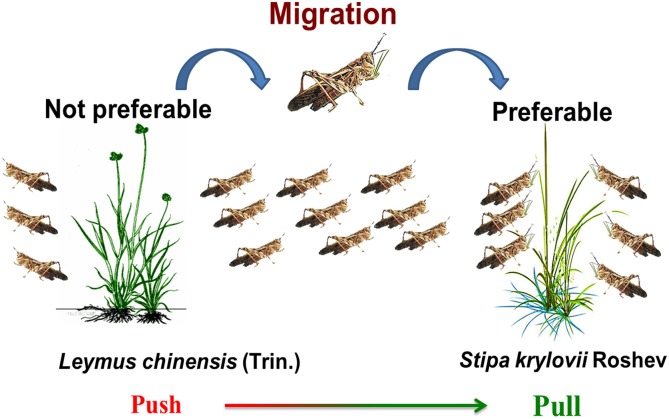
Illustration of the ‘push-pull’ roles of *L. chinensis* and *S. krylovii* to *O. asiaticus* migration and plague.

Based on those researches that focused on plant associations and grasshopper performance or distribution, we hypothesized that *S. krylovii*, acts as the ‘pull,’ and the *L. chinensis*, as a ‘push,’ which contributes to grasshopper migration and consequently aggravates plague outbreaks in *S. krylovii*-dominated grassland (Figure 12). Particularly under the background of climate change (Stige et al., 2007), those areas of *Stipa*-dominated grasslands are the potential regions of *O. asiaticus* expansion or plague, such as the northern China, Siberia, Mongolia, Kazakhstan (Chen and Wang, 2000; Han et al., 2008). So, the monitoring and control of grasshopper *O. asiaticus* should be strengthened in those areas. Besides, the opposite function and role of these two grasses implied that the ‘push-pull’ strategy (Cook et al., 2007) would be a potential management tool to control future grasshopper outbreak.

The reasons underpinning the buildup of insect populations can be related to chemical traits of plants, such as the presence of important nutrients and plant secondary metabolites (Mendelsohn and Balick, 1995; Raymond et al., 2004). Available protein, carbohydrate and lipid content are important for insect herbivores growth (Simpson et al., 2004). They have well-defined nutritional requirements (Behmer, 2009), and generally prefer plants with suitable nutritional qualities, as the optimal food (Bernays et al., 1994; Powell et al., 2006). The availability of such plants may increase the probability of pest population outbreaks. Plant secondary metabolites, such as tannins, phenols, flavonoids, alkaloids, terpenoids, and glucosinolates, generally function as toxins or repellents (Despres et al., 2007). Those metabolites are detrimental to insect growth (Bernays and Chapman, 1994; Pérez et al., 2003; Unsicker et al., 2008). So, reducing access to key nutrients or increased levels of secondary compounds may decrease the probability of pest population outbreaks (Simpson et al., 2004; Cease et al., 2012). In the present study, we found that grasshopper density was positively related to nutrition content, but negatively related to secondary metabolites, which suggested that grasshopper plague events were confined to habitats providing high nutrition and low toxin levels whereby growth and development is optimal. These results are also supported by the general hypothesis that nutritious habitats benefit insect growth, but plant secondary compounds have detrimental effects on growth (Behmer, 2009; Despres et al., 2007; Wetzel et al., 2016). Dietary stress resulting from feeding on plants containing high levels of secondary metabolites could well explain the poor growth performance and low distribution of grasshopper, *O. asiaticus*, when confronted with *L. chinensis*, consequently resulting in the push role of *L. chinensis*. In contrast, *S. krylovii*, a preferred host species containing few secondary metabolites, benefited grasshopper growth and plague, and consequently acting as the pull.

Gene expression and related enzyme function were the underpinning mechanism of insect performance variations (Bishop and Guarente, 2007; Taguchi and White, 2008; Roy et al., 2016). Diet stress from plant chemical exposure can also result in different gene expression of insect herbivores (Badisco et al., 2013; Roy et al., 2016; Huang et al., 2017b). For example, the gene expression patterns of digestive and detoxifying enzymes, immunity, transporters, and peritrophic membrane associated transcripts varied significantly in *Spodoptera* spp when confronted with different diet stress (Janz and Nylin, 2008; Ragland et al., 2015; Roy et al., 2016). Those changed genes were the basis of genetic adaptation, and allowed the rapid induction of arrays of broader or more robustly active digestive or detoxifying enzymes in herbivores after the consumption of toxic plants (Bishop and Guarente, 2007; Despres et al., 2007). Such as, the gene expression and activity of CYP450s, glutathione-*S*-transferase, and carboxylesterase were generally positively correlated to levels of secondary plant metabolites (Despres et al., 2007; Roy et al., 2016). These rapid biochemical responses to diet stress from changing plant chemical traits are vital for insect survival and growth. From our previous study (Huang et al., 2017b), we also found that grasshoppers feeding on *L. chinensis* had high gene expression and enzyme activity associated with detoxification, which implies that grasshopper survival requires greater consumption to detoxify these compounds and consequently resulting in reduced phenotypic parameters (Karban and Agrawal, 2002; Castañeda et al., 2010), such as size and growth rate compared to grasshoppers feeding on a suitable host species. For example, *S. krylovii* has lower levels of secondary metabolites, grasshoppers feeding on this plant produce fewer detoxifying enzymes, and was required to expend less excess energy to survive. Consequently, the higher survival and growth rates contributed to plague outbreaks in *S. krylovii*-dominated grassland.

**FIGURE 13:**
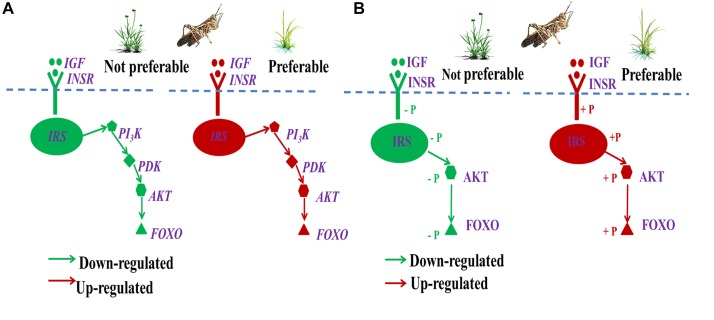
Regulation of gene expression **(A)** and protein phosphorylation level **(B)** of ILP (IGF → FOXO cascade) during different diet stress. IGF, insulin-like growth factor; INSR, homologous insulin receptor; IRS, insulin receptor substrate; PI_3_K, phosphoinositide 3-kinase; PDK, 3-phosphoinositide-dependent protein kinase; AKT, protein kinase B; and FOXO, forkhead transcription factor.

Gene expression of detoxification was mainly regulated by the ILP signaling pathway IGF → FOXO (Wolkow et al., 2002; Wu and Brown, 2006; Badisco et al., 2013). The high phosphorylation level of FOXO generally down-regulated the expression of detoxification-related genes, with dephosphorylated FOXO (low phosphorylation level) having the opposite effect (Bishop and Guarente, 2007; Kramer et al., 2008; Taguchi and White, 2008; Hedrick, 2009; Ragland et al., 2015). We found that high levels of secondary metabolites in *L. chinensis* significantly down-regulated gene expression and phosphorylation of the IGF → FOXO cascade (Figure 13), which could promote gene expression of detoxification enzymes in *O. asiaticus* that fed on *L. chinensis*. In addition, the significantly down-regulated genes, IGF/INSR/IRS/PI3K/PDK/AKT, could also down-regulate growth-related gene expression, which is also generally detrimental to insect growth. The down-regulated ILP indicated poor adaptation of grasshopper to *L. chinensis*. These important gene variations revealed why grasshoppers prefer *S. krylovii*, and avoid *L. chinensis*.

## Conclusion

In conclusion, the grass *L. chinensis* contains high levels of secondary metabolites that down-regulated the ILP signaling pathway, resulting in the poor growth of *O. asiaticus* grasshopper, consequently driving migration to *S. krylovii*-dominated grassland. Therefore, we conclude that grasshoppers have an intelligent compromise to energy demand and detoxification cost, and propose a hypothesis that dietary stress from secondary metabolites contributes to grasshopper, *O. asiaticus*, migration, and plague outbreaks by regulating insect ILP.

## Ethics Statement

Insects (*O. asiaticus*) were collected from the Xilin Gol grassland from 2011 to 2017. It is a common agricultural pest and not in the “List of Protected Animals in China.” No permits were required for the described field studies.

## Author Contributions

ZZ and XH designed the experiments. XH, SuL, and JM performed the experiments. XH, WL, SeL, and XT analyzed the data. XH, SuL, and MM wrote the manuscript. All authors reviewed and considered the manuscript.

## Conflict of Interest Statement

The authors declare that the research was conducted in the absence of any commercial or financial relationships that could be construed as a potential conflict of interest.

## References

[B1] Badisco L. Wielendaele P. V. Broeck J. V. (2013). Eat to reproduce: a key role for the insulin signaling pathway in adult insects. *Front. Physiol.* 4:202. 10.3389/fphys.2013.00202 23966944 PMC3735985

[B2] Baldwin I. T. (1998). Jasmonate-induced responses are costly but benefit plants under attack in native populations. *Proc. Natl. Acad. Sci. U.S.A.* 95 8113–8118. 10.1073/pnas.95.14.8113 9653149 PMC20938

[B3] Bawa S. F. Yadav S. P. (1986). Protein and mineral contents of green leafy vegetables consumed by Sokoto population. *J. Sci. Food Agric.* 37 504–506. 10.1002/jsfa.2740370512

[B4] Becerra J. X. (2003). Synchronous coadaptation in an ancient case of herbivory. *Proc. Natl. Acad. Sci. U.S.A.* 100 12804–12807. 10.1073/pnas.2133013100 14555762 PMC240699

[B5] Behmer S. T. (2009). Insect herbivore nutrient regulation. *Annu. Rev. Entomol.* 54 165–187. 10.1146/annurev.ento.54.110807.090537 18764740

[B6] Bernays E. A. Bright K. L. Gonzalez N. Angel J. (1994). Dietary mixing in a generalist herbivore: tests of two hypotheses. *Ecology* 75 1997–2006. 10.2307/1941604

[B7] Bernays E. A. Chapman R. F. (1994). *Host-Plant Selection by Phytophagous Insects.* Boston, MA: Springer.

[B8] Bishop N. A. Guarente L. (2007). Genetic links between diet and lifespan: shared mechanisms from yeast to humans. *Nat. Rev. Genet.* 8 835–844. 10.1038/nrg2188 17909538

[B9] Castañeda L. E. Figueroa C. C. Fuentes-Contreras E. Niemeyer H. M. Nespolo R. F. (2010). Physiological approach to explain the ecological success of ‘superclones’ in aphids: interplay between detoxification enzymes, metabolism and fitness. *J. Insect Physiol.* 56 1058–1064. 10.1016/j.jinsphys.2010.02.019 20223246

[B10] Cease A. J. Elser J. J. Ford C. F. Hao S. G. Kang L. Harrison J. F. (2012). Heavy livestock grazing promotes locust outbreaks by lowering plant nitrogen content. *Science* 335 467–469. 10.1126/science.1214433 22282812

[B11] Chen Z. Z. Wang S. P. (2000). *Typical Steppe Ecosystems of China.* Beijing: Science Press.

[B12] Claeys I. Simonet G. Poels J. Van Loy T. Vercammen L. DeLoof A. (2002). Insulin-related peptides and their conserved signal transduction pathway. *Peptides* 23 807–816. 10.1016/S0196-9781(01)00666-0 11897402

[B13] Cook S. M. Khan Z. R. Pickett J. A. (2007). The use of push-pull strategies in integrated pest management. *Ann. Rev. Entomol.* 52 375–400. 10.1146/annurev.ento.52.110405.091407 16968206

[B14] Despland E. Simpson S. J. (2005). Food choices of solitarious and gregarious grasshoppers reflect cryptic and aposematic antipredator strategies. *Anim. Behav.* 69 471–479. 10.1016/j.anbehav.2004.04.018

[B15] Despres L. David J. P. Gallet C. (2007). The evolutionary ecology of insect resistance to plant chemicals. *Trends Ecol. Evol.* 22 298–307. 10.1016/j.tree.2007.02.010 17324485

[B16] Dicke M. Baldwin I. T. (2010). The evolutionary context for herbivore-induced plant volatiles: beyond the ‘cry for help’. *Trends Plant Sci.* 15 167–175. 10.1016/j.tplants.2009.12.002 20047849

[B17] Dicke M. Loon J. J. A. (2000). Multitrophic effects of herbivore-induced plant volatiles in an evolutionary context. *Entomol. Exp. Appl.* 97 237–249. 10.1023/A:1004111624780

[B18] Dussourd D. E. (2003). Chemical stimulants of leaf-trenching by cabbage loopers: natural products, neurotransmitters, insecticides, and drugs. *J. Chem. Ecol.* 29 2023–2047. 10.1023/A:1025630301162 14584674

[B19] Franzke A. Unsicker S. B. Specht J. (2010). Being a generalist herbivore in a diverse world: how do diets from different grasslands influence food plant selection and fitness of the grasshopper *Chorthippus parallelus*. *Ecol. Entomol.* 35 126–138. 10.1111/j.1365-2311.2009.01168.x

[B20] Friedman M. Levin C. E. Choi S. H. Kozukue E. Kozukue N. (2006). HPLC analysis of catechins, theaflavins, and alkaloids in commercial teas and green tea dietary supplements: comparison of water and 80% ethanol/water extracts. *J. Food Sci.* 71 328–337. 10.1111/j.1750-3841.2006.00090.x

[B21] Fujisawa T. Hayakawa E. (2012). Peptide signaling in Hydra. *Int. J. Dev. Biol.* 56 543–550. 10.1387/ijdb.113477tf 22689368

[B22] Gatehouse J. A. (2002). Plant resistance towards insect herbivores: a dynamic interaction. *New Phytol.* 156 145–169. 10.1046/j.1469-8137.2002.00519.x 33873279

[B23] Giri A. P. Wünsche H. Mitra S. Zavala J. A. Muck A. Svatos A. (2006). Molecular interactions between the specialist herbivore *Manduca sexta* (Lepidoptera, Sphingidae) and its natural host *Nicotiana attenuata*. VII. changes in the plant’s proteome. *Plant Physiol.* 142 1621–1641. 10.1104/pp.106.088781 17028148 PMC1676057

[B24] Griffin S. Wyllie S. G. Markham J. (1999). Determination of octanol-water partition coefficient for terpenoids using reversed-phase high-performance liquid chromatography. *J. Chromatogr. A* 864 221–228. 10.1016/S0021-9673(99)01009-2 10669289

[B25] Guo J. L. (2007). Determination of tannic acid in *Phyllanthus emblica* l. by high performance liquid chromatography. Chinese. *J. Spectrosc. Lab.* 24 911–913. 10.1004/8138200705-0911-03

[B26] Guo Z. W. Li H. C. Gan Y. L. (2006). Grasshopper (Orthoptera: Acrididae) biodiversity and grassland ecosystems. *Insect Sci.* 13 221–227. 10.1111/j.744-7917.2006.00086.x

[B27] Han J. G. Zhang Y. J. Wang C. J. Wang Y. R. Han G. D. (2008). Rangeland degradation and restoration management in China. *Rangeland J.* 30 233–239. 10.1071/RJ08009-1036-9872/08/020233 26208005

[B28] Hedrick S. M. (2009). The cunning little vixen: foxo and the cycle of life and death. *Nat. Immunol.* 10 1057–1063. 10.1038/ni.1784 19701188 PMC2856448

[B29] Helmus M. R. Dussourd D. E. (2005). Glues or poisons: which triggers vein cutting by monarch caterpillars? *Chemoecology* 15 45–49. 10.1007/s00049-005-0291-y

[B30] Howe G. A. Jander G. (2008). Plant immunity to insect herbivores. *Annu. Rev. Plant Biol.* 59 41–66. 10.1146/annurev.arplant.59.032607.092825 18031220

[B31] Huang X. Mcneill M. R. Zhang Z. (2016). Quantitative analysis of plant consumption and preference by *Oedaleus asiaticus* (Acrididae: Oedipodinae) in changed plant communities consisting of three grass species. *Environ. Entomol.* 45 163–170. 10.1093/ee/nvv172 26577863

[B32] Huang X. B. Mcneill M. R. Ma J. C. Qin X. H. Tu X. B. Cao G. C. (2017a). Biological and ecological evidences suggest *Stipa krylovii* (Pooideae), contributes to optimal growth performance and population distribution of the grasshopper *Oedaleus asiaticus*. *Bull. Entomol. Res.* 107 401–409. 10.1017/S000748531600105X 28137319

[B33] Huang X. B. Ma J. C. Qin X. H. Tu X. B. Cao G. C. Wang G. J. (2017b). Biology, physiology and gene expression of grasshopper *Oedaleus asiaticus* exposed to diet stress from plant secondary compounds. *Sci. Rep.* 7:8655. 10.1038/s41598-017-09277-z 28819233 PMC5561062

[B34] Huang X. B. Wu H. H. Qin X. H. Cao G. C. Wang G. J. Nong X. Q. (2015). Comprehensive evaluation and risk assessment of grasshoppers’ habitat based on a projection pursuit model. *Acta Pratacult. Sin.* 24 25–33. 10.11686/cyxb20150504

[B35] Ibanez S. Manneville O. Miquel C. Taberlet P. Valentini A. Aubert S. (2013). Plant functional traits reveal the relative contribution of habitat and food preferences to the diet of grasshoppers. *Oecologia* 173 1459–1470. 10.1007/s00442-013-2738-0 24096738

[B36] Janz N. Nylin S. (2008). “The oscillation hypothesis of host-plant range and speciation,” in *Specialization, Speciation, and Radiation: The Evolutionary Biology of Herbivorous Insects*, ed. Tilmon K. J. (Berkeley, CA: University of California Press), 203–215. 10.1525/california/9780520251328.003.0015

[B37] Ji R. Xie B. Y. Li D. M. Yuan H. Yang H. S. (2007). Effects of reed population pattern on spatial distribution of *Locusta migratoria manilensis* in Nandagang wetland. *Chin. Bull. Entomol.* 46 830–833.

[B38] Karban R. Agrawal A. A. (2002). Herbivore offense. *Ann. Rev. Ecol. Syst.* 33 641–664. 10.1146/annurev.ecolsys.33.010802.150443

[B39] Kawada T. Sekiguchi T. Sakai T. Aoyama M. Satake H. (2010). Neuropeptides, hormonepeptides, and their receptors in *Cionaintestinalis*: an update. *Zool. Sci.* 27 134–153. 10.2108/zsj.27.134 20141419

[B40] Kessler A. Baldwin I. T. (2002). Plant responses to insect herbivory: the emerging molecular analysis. *Annu. Rev. Plant Biol.* 53 299–328. 10.1146/annurev.arplant.53.100301.135207 12221978

[B41] Kramer J. M. Slade J. D. Staveley B. E. (2008). Foxo is required for resistance to amino acid starvation in *Drosophila*. *Genome* 51 668–672. 10.1139/G08-04 18650956

[B42] Liu G. H. Wang G. J. Wang S. P. Han J. G. Wang X. R. Hao S. G. (2013). The diet composition and trophic niche of main herbivores in the Inner Mongolia desert steppe. *Acta Agrestia Sin.* 33 856–866. 10.5846/stxb201207281071

[B43] Masloski K. Greenwood C. Reiskind M. Payton M. (2014). Evidence for diet-driven habitat partitioning of melanoplinae and gomphocerinae (Orthoptera: Acrididae) along a vegetation gradient in a Western Oklahoma Grassland. *Environ. Entomol.* 43 1209–1214. 10.1603/EN13349 25203904

[B44] Mendelsohn R. Balick M. J. (1995). The value of undiscovered pharmaceuticals in tropical forests. *Econ. Bot.* 49 223–228. 10.1007/BF02862929

[B45] Musser R. O. Hum-Musser S. M. Eichenseer H. Peiffer M. Ervin G. Murphy J. B. (2002). Herbivory: caterpillar saliva beats plant defences. *Nature* 416 599–600. 10.1038/416599a 11948341

[B46] Naczk M. (2004). Extraction and analysis of phenolics in food. *J. Chromatogr. A* 1054 95–111. 10.1016/s0021-9673(04)01409-8 15553136

[B47] Ossipov V. Nurmi K. Loponen J. Prokopiev N. Haukioja E. Pihlaja K. (1995). HPLC isolation and identification of flavonoids from white birch betula pubescens, leaves. *Biochem. Syst. Ecol.* 23 213–222. 10.1016/0305-1978(94)00092-u

[B48] Padul M. V. Tak R. D. Kachole M. S. (2012). Protease inhibitor (PI) mediated defense in leaves and flowers of pigeonpea (protease inhibitor mediated defense in pigeonpea). *Plant Physiol. Biochem.* 52 77–82. 10.1016/j.plaphy.2011.10.018 22305069

[B49] Pérez H. Díaz S. Vendramini F. Cornelissen J. H. C. Gurvich D. E. Cabido M. (2003). Leaf traits and herbivore selection in the field and in cafeteria experiments. *Aust. Ecol.* 28 642–665. 10.1046/j.1442-9993.2003.01321.x

[B50] Powell G. Tosh C. R. Hardie J. (2006). Host plant selection by aphids: behavioral, evolutionary, and applied perspectives. *Ann. Rev. Entomol.* 51 309–330. 10.1146/annurev.ento.51.110104.151107 16332214

[B51] Qin X. Hao K. Ma J. Huang X. Tu X. Ali M. P. (2017). Molecular ecological basis of grasshopper (*Oedaleus asiaticus*) phenotypic plasticity under environmental selection. *Front. Physiol.* 8:770. 10.3389/fphys.2017.00770 29066978 PMC5641302

[B52] Ragland G. J. Almskaar K. Vertacnik K. L. Gough H. M. Feder J. L. Hahn D. A. (2015). Differences in performance and transcriptome-wide gene expression associated with Rhagoletis (Diptera:Tephritidae) larvae feeding in alternate host fruit environments. *Mol. Ecol.* 24 2759–2776. 10.1111/mec.13191 25851077

[B53] Raubenheimer D. Simpson S. J. (2004). Nutrient balancing in grasshoppers: behavioural and physiological correlates of dietary breadth. *J. Exp. Biol.* 206 1669–1681. 10.1242/jeb.00336 12682099

[B54] Raymond B. V. David N. K. Zhong C. (2004). Performance of a generalist grasshopper on a C3 and a C4 grass: compensation for the effects of elevated CO2 on plant nutritional quality. *Oecologia* 140 96–103. 10.1007/s00442-004-1555-x 15069636

[B55] Rominger A. J. Miller T. E. X. Collins S. L. (2009). Relative contributions of neutral and niche-based processes to the structure of a desert grassland grasshopper community. *Oecologia* 161 791–800. 10.1007/s00442-009-1420-z 19629531

[B56] Roy A. Walker W. B. Vogel H. Chattington S. Larsson M. C. Anderson P. (2016). Diet dependent metabolic responses in three generalist insect herbivores *Spodoptera* spp. *Insect Biochem. Molec.* 71 91–105. 10.1016/j.ibmb.2016.02.006 26908076

[B57] Schutz S. Weißbecker B. Klein A. Hummel H. E. (1997). Host plant selection of the Colorado potato beetle as influenced by damage induced volatiles of the potato plant. *Naturwissenschaften* 84 212–217. 10.1007/s001140050381

[B58] Scriber J. M. (2002). Evolution of insect-plant relationships: chemical constraints, coadaptation, and concordance of insect/plant traits. *Entomol. Exp. Appl.* 104 217–235. 10.1023/A:1021292604205

[B59] Sim C. Denlinger D. L. (2008). Insulin signaling and FOXO regulate the overwintering diapause of the mosquito *Culex pipiens*. *Proc. Natl. Acad. Sci. U.S.A.* 105 6777–6781. 10.1073/pnas.0802067105 18448677 PMC2373331

[B60] Simpson S. J. Sibly R. M. Lee K. P. Behmer S. T. Raubenheimer D. (2004). Optimal foraging when regulating intake of multiple nutrients. *Anim. Behav.* 68 1299–1311. 10.1016/j.anbehav.2004.03.003

[B61] Stige L. C. Chan K. S. Zhang Z. B. Frank D. Stenseth N. C. (2007). Thousand-year-long Chinese time series reveals climatic forcing of decadal locust dynamics. *Proc. Natl. Acad. Sci. U.S.A.* 104 16188–16193. 10.1073/pnas.0706813104 17878300 PMC2042183

[B62] Taguchi A. White M. F. (2008). Insulin-like signaling, nutrient homeostasis, and life span. *Annu. Rev. Physiol.* 70 191–212. 10.1146/annurev.physiol.70.113006.100533 17988211

[B63] Unsicker S. B. Franzke A. Specht J. Köhler G. Linz J. Renker C. (2010). Plant species richness in montane grasslands affects the fitness of a generalist grasshopper species. *Ecology* 91 1083–1091. 10.1890/09-0402.1 20462122

[B64] Unsicker S. B. Oswald A. Kohler G. Weisser W. W. (2008). Complementarity effects through dietary mixing enhance the performance of a generalist insect herbivore. *Oecologia* 156 313–324. 10.1007/s00442-008-0973-6 18301924 PMC2757592

[B65] Wetzel W. C. Kharouba H. M. Robinson M. Holyoak M. Karban R. (2016). Variability in plant nutrients reduces insect herbivore performance. *Nature* 539 425–427. 10.1038/nature20140 27749815

[B66] Whitman D. W. (1990). “Grasshopper chemical communication,” in *Biology of Grasshoppers*, eds Chapman R. F. Joern A. (New York, NY: John Wiley and Sons), 357–391.

[B67] Wolkow C. A. Muñoz M. J. Riddle D. L. Ruvkun G. (2002). Insulin receptor substrate and p55 orthologous adaptor proteins function in the *Caenorhabditis elegans* daf-2/insulin-like signaling pathway. *J. Biol. Chem.* 277 49591–49597. 10.1074/jbc.M207866200 12393910

[B68] Wu H. H. Xu Y. H. Cao G. C. Gexigedu R. Liu Z. Y. He B. (2012). Ecological effects of typical grassland types in inner mongolia on grasshopper community. *Sci. Agric. Sin.* 45 4178–4186.

[B69] Wu J. Q. Baldwin I. T. (2010). New insights into plant responses to the attack from insect herbivores. *Annu. Rev. Genet.* 44 1–24. 10.1146/annurev-genet-102209-163500 20649414

[B70] Wu Q. Brown M. R. (2006). Signaling and function of insulin-like peptides in insects. *Annu. Rev. Entomol.* 51 1–24. 10.1146/annurev.ento.51.110104.151011 16332201

[B71] Zavala J. A. Patankar A. G. Gase K. Baldwin I. T. (2004). Constitutive and inducible trypsin proteinase inhibitor production incurs large fitness costs in, *Nicotiana attenuata*. *Proc. Natl. Acad. Sci. U.S.A.* 101 1607–1612. 10.1073/pnas.0305096101 14757829 PMC341788

[B72] Zhang M. C. Fielding D. J. (2011). Populations of the northern grasshopper, *Melanoplus borealis* (Orthoptera: Acrididae), in Alaska are rarely food limited. *Environ*. *Entomol.* 40 541–548. 10.1603/EN10179 22251631

[B73] Zhang W. Z. He B. Cao G. C. Zhang Z. H. Wu Y. H. Liu S. C. (2013). Quantitative analysis of the effects of *Stipa krylovii* and *Leymus chinensis* on *Odaleous asiaticus*. *Acta Pratacult. Sin.* 22 302–309. 27562455

[B74] Zhang Z. J. Elser J. J. Cease A. J. Zhang X. M. Yu Q. (2014). Grasshoppers regulate N:P stoichiometric homeostasis by changing phosphorus contents in their frass. *PLoS One* 9:e103697. 10.1371/journal.pone.0103697 25089521 PMC4121213

[B75] Zhu E. L. (2004). *The Occurrence and Management of Locusta Migratoria Manilensis in Chinese.* Beijing: China Agricultural Press.

