# Supplementary material for: Dietary Stress From Plant Secondary Metabolites Contributes to Grasshopper (Oedaleus asiaticus) Migration or Plague by Regulating Insect Insulin-Like Signaling Pathway
Source: Front Physiol. 2019 May 3;10:531. doi: 10.3389/fphys.2019.00531 (PMC6509742; doi:10.3389/fphys.2019.00531)
Supplement: Supplementary file 1 [file Table_1.DOCX]

**Table S1 Designed sequences of qRT-PCR primers for the seven genes**

| Gene name | Primers | Primer sequences (5’-3’) |
| --- | --- | --- |
| β-actin | Forward | CCCATCTATGAAGGTTACGC |
|  | Reverse | CTTGATGTCACGGACGATTT |
| *IGF* | Forward | AAACCCGGAGAACTGCACAAATGC |
|  | Reverse | TGCCGATAAGATTGTTATGAGCAAGG |
| *INSR* | Forward | TTATTATCGGAAAGGCTCCAAAGG |
|  | Reverse | CACTACACCGTAACTCCAGACATCG |
| *IRS* | Forward | ATCCCGATCCAGAACCAGATGC |
|  | Reverse | AAGACGATGCCGACAAAGAGCC |
| *PI3K* | Forward | TATACAAGTCTGTAACATCCCACG |
|  | Reverse | ACATCTCCTGCTTTGAGTCCTT |
| *PDK* | Forward | GGAGTTTAGGGTGCATCTTGTA |
|  | Reverse | GTATTGCCACCTCTTGGTTAGT |
| *AKT* | Forward | GGTAATGCCTCGTAATTTCTGT |
|  | Reverse | AACTCTTTATGATGGGTCTTCT |
| *FOXO* | Forward | CATCACGCAGGCCATCCAG |
|  | Reverse | GCTGTTGCTGTCGCCCTTG |
