# Supplementary material for: Dietary Stress From Plant Secondary Metabolites Contributes to Grasshopper (Oedaleus asiaticus) Migration or Plague by Regulating Insect Insulin-Like Signaling Pathway
Source: Front Physiol. 2019 May 3;10:531. doi: 10.3389/fphys.2019.00531 (PMC6509742; doi:10.3389/fphys.2019.00531)
Supplement: Supplementary file 2 [file Table_2.DOCX]

**Table S2 ELISA reagents**

| **Items** | **Materials** | **48 tests** |
| --- | --- | --- |
| 1 | Microelisa Stripplate | 12×4 Strips |
| 2 | Standards×6 vials | 0.5ml×6 vials |
| 3 | Sample Diluent | 3.0ml |
| 4 | HRP-Conjugate Reagent | 5.0ml |
| 5 | 20×Wash Solution | 15ml |
| 6 | Stop Solution | 3.0ml |
| 7 | Chromogen Solution A | 3.0ml |
| 8 | Chromogen Solution B | 3.0ml |
| 9 | Closure Plate Membrane | 2 |
| 10 | Sealed Bags | 1 |
